# Logistics of defense: The contribution of endomembranes to plant innate immunity

**DOI:** 10.1083/jcb.202307066

**Published:** 2024-03-29

**Authors:** Deepak D. Bhandari, Federica Brandizzi

**Affiliations:** 1 MSU-DOE Plant Research Laboratory, Michigan State University, East Lansing, MI, USA; 2 Great Lakes Bioenergy Research Center, Michigan State University, East Lansing, MI, USA; 3 Department of Plant Biology, Michigan State University, East Lansing, MI, USA

## Abstract

Phytopathogens cause plant diseases that threaten food security. Unlike mammals, plants lack an adaptive immune system and rely on their innate immune system to recognize and respond to pathogens. Plant response to a pathogen attack requires precise coordination of intracellular traffic and signaling. Spatial and/or temporal defects in coordinating signals and cargo can lead to detrimental effects on cell development. The role of intracellular traffic comes into a critical focus when the cell sustains biotic stress. In this review, we discuss the current understanding of the post-immune activation logistics of plant defense. Specifically, we focus on packaging and shipping of defense-related cargo, rerouting of intracellular traffic, the players enabling defense-related traffic, and pathogen-mediated subversion of these pathways. We highlight the roles of the cytoskeleton, cytoskeleton–organelle bridging proteins, and secretory vesicles in maintaining pathways of exocytic defense, acting as sentinels during pathogen attack, and the necessary elements for building the cell wall as a barrier to pathogens. We also identify points of convergence between mammalian and plant trafficking pathways during defense and highlight plant unique responses to illustrate evolutionary adaptations that plants have undergone to resist biotic stress.

## Teaser

Bhandari and Brandizzi review the trafficking pathways recruited upon plant defense activation and how these pathways are targeted by pathogens to dampen immunity and cell wall fortifications resulting from immune activation.

## Introduction

Plant cells coexist with a mixture of beneficial and pathogenic microbes. Thus, to limit ectopic defense activation while balancing the allocation of resources between defense and growth, plants have evolved a sophisticated surveillance system and rapid immune responses. Successful defense response against phytopathogens (herein pathogens) is the culmination of innumerable battles possibly originated by diverse pathogens at different sites within and outside the plant cell. Plant cells achieve this through a multitiered immune system, which, upon pathogen recognition results in a massive redeployment of cytoskeleton and endomembrane activities to limit pathogen growth. In turn, pathogens have evolved to target various endomembrane organelles and trafficking pathways of the host to subvert and/or dampen immune signaling and enable pathogen proliferation (Nomura et al., 2006; Guo et al., 2016; Petre et al., 2021; Bhandari et al., 2023).

Pathogens reside in different spaces in the plant. For example, bacterial pathogens like *Pseudomonas* and *Xanthomonas* live outside the cell in the apoplast, while some filamentous pathogens (e.g., oomycetes and fungi) penetrate the host cell, and some pathogens (e.g., *Ralstonia* and certain *Xanthomonas* species) colonize the plant vasculature (Xin et al., 2018; Wang et al., 2021a; Kemen and Jones, 2012; Wang et al., 2019b; Peeters et al., 2013) (Box 1 and Box 2). Whether residing in extracellular spaces or invading cells, pathogens establish physical contact with host cells to either dampen immune response or to facilitate nutrient uptake with the goal of enabling their own growth. Phytopathogens can achieve this by different means, such as injecting effectors, for example, phytotoxins and virulence factors into the host cell, to hijack host processes, including trafficking (Xin et al., 2018; Hrmova and Gilliham, 2018; Robin et al., 2018). Hence, studying intracellular trafficking in plant cells under pathogen attack will provide important insights into both plant defense and pathogen virulence mechanisms, and it will enable the identification of strategies and targets of intervention aimed at minimizing plant diseases induced by pathogens.

Box 1 Plant immunity concepts 101

1 Adapted versus non-adapted pathogen—An adapted pathogen can infect a plant, be virulent, and be able to grow and spread. The plant species that can be infected by a pathogen is classified as a host plant, and the resulting interaction is known as a compatible interaction. Most pathogens have a limited range of hosts. A non-adapted pathogen is any microbe that attempts to colonize a plant outside its host range. Thus, the same pathogen can be classified as adapted or non-adapted based on the plant species under study.
2 MAMPs/PAMPs/DAMPs—Conserved microbe-/pathogen-/damage-associated molecular patterns which are recognized by cell-surface pattern recognition receptors (PRRs). Examples include bacterial flagellin, fungal cell wall chitin, glucans, or plant cell wall polysaccharides released due to physical damage or herbivore feeding.
3 PTI—Pattern-triggered immunity. Immunity triggered by the recognition of MAMPs/DAMPs by PRRs, resulting in the activation of immune signaling cascades, accumulation of SA, and secretion of antibacterial proteins.
4 ETI—Effector-triggered immunity. Immunity triggered by intracellular recognition of pathogen secreted effectors by plant nucleotide-binding leucine-rich repeat proteins (NLRs). ETI is usually activated when plants combat incompatible pathogen strains. Activation of ETI results in faster and stronger activation of immune signaling cascades, large-scale transcriptional reprogramming, accumulation of SA, secretion of antibacterial proteins, and in some cases localized cell death. Both PTI and ETI share signaling networks and differ largely in their speed and amplitude.
5 Basal immunity—A defense response that is elicited upon recognition of an adapted pathogen which results in slowing down pathogen infection. Basal immunity comprises aspects of PTI and pathogen-mediated susceptibility.

Box 2 Focal immunity

Diverse filamentous pathogens (fungi, oomycetes) with the capacity to form haustoria lead to the formation of the extrahaustorial matrix (EHM) interface with their plant hosts. A general strategy adopted by filamentous pathogens is to hijack host membrane trafficking components to dampen defense and facilitate nutrient uptake. Biotrophic filamentous pathogens use a specialized infection structure known as the haustorium. Plants defend themselves against filamentous pathogens by deploying polarized defense to the site of penetration by the hyphae. This process is broadly defined as focal immunity. Membrane trafficking components play a crucial role in limiting pathogen penetration by enabling focal immunity. Focalized defense encompasses re-routing and relocalization of host trafficking components such as ER and Golgi apparatus close to the EHM and results in focalized secretion of defense-related compounds and CW reinforcements. The contributing factors to the biogenesis of EHM are not fully understood; however, EHM is derived from the host cells and its composition is different from the PM. Similarities exist between plant and mammalian defense responses against filamentous pathogens. The EHM is also devoid of receptors and proteins usually populating the PM; thus, the plant EHM is similar to the perimicrobial interface observed during mammalian–pathogen infection. The membrane-bound Arabidopsis synaptogamin1 (SYT1), which is associated with the ER–PM contact sites (EPCS), is also found on the EHM microdomains, underpinning the localization of ER to the site of pathogen invasion. The Rab GTPase, Rab7 is found on both the plant EHM (infected with *P. infestans*) and the mammalian perimicrobial interface (infected with *Salmonella enterica*), underscoring the importance of membrane trafficking components in both plant and mammalian immunity. Evidence from different pathogen–host interfaces supports the recruitment of different Rabs by the host immune system based on the pathogen encountered. In addition to the Rab GTPases, plant SNAREs, such as SYP121 (PEN1) also play an important role in limiting penetration. Nonetheless, the plant CW provides a stark point of defense between mammalian and plant defense responses. Upon pathogen attack, the CW is reinforced with many appositions. While a comprehensive understanding of CW fortification against different filamentous pathogens is yet to be determined, some CW polysaccharides are known to be involved in CW-directed defense. For example, AGPs deposited into the CW upon *Pst* infection are also found on the EHM upon oomycete infection, suggesting that some aspects of CW-directed defense may be conserved.

In this review, using examples chiefly from bacterial and fungal pathogens, we highlight findings that explain how the plant intracellular trafficking pathways are repurposed upon immune activation, with a specific focus on pathways of exocytic defense for the secretion of antimicrobial compounds, a possible role of the cytoskeleton as a sentinel of pathogen attack, and cell wall (CW) remodeling as part of plant defense strategies. We also highlight the current understanding of pathogen effectors that target host trafficking pathways.

### Intracellular trafficking in plants: A brief overview

**Figure 1.**
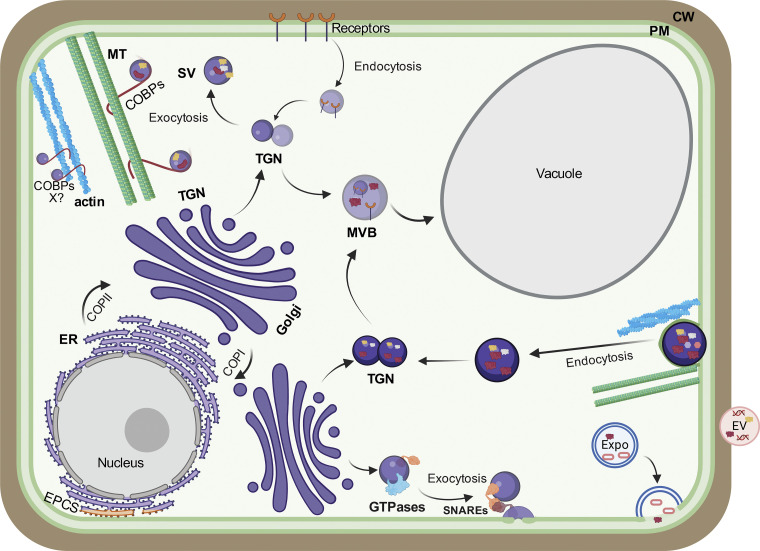
**An overview of plant intracellular trafficking pathways and organelles.** In plant cells, biosynthetic traffic is coordinated by both the conventional and unconventional trafficking pathways. Newly synthesized proteins are cotranslationally folded in the ER for transport to the Golgi apparatus via COPII traffic. Proteins and lipids are transported back to the ER from the Golgi via the retrogade COPI pathway. Cargo matures through the cis-, medial-, and trans*-*Golgi where posttranslational modifications are added before loading into secretory vesicles at the TGN. Vesicles are sorted at the TGN, which acts as a sorting hub for endocytic and exocytic traffic, and transported to their final destination—PM, MVB, or the vacuole. The plant TGN also acts as the early endosome. Secretory vesicles (SV) are transported to their destination over the actin (blue) and MT (green) cytoskeleton. The cytoskeleton also enables endocytosis of the vesicles from the PM. Vesicle transport across the cell is aided by small GTPases that act as molecular on/off switches enabling transport, tethering, and fusion of vesicles. Vesicle fusion to the PM is mediated by soluble N-ethylmalemide-SNAREs, which determine the fidelity of vesicle fusion to the exocyst complex at its appropriate destination. EXPOs are double-membrane structures, which form a part of unconventional trafficking in plants. EXPO fuses to the PM releasing vesicles into the CW. EV are lipid membrane–bound vesicles released into the extracellular space. EV can contain small RNAs and defense-related proteins and can be endocytosed by pathogens leading to reduced pathogen virulence. A new class of proteins termed COBPs, which link vesicles with the cytoskeleton, are emerging key players in endocytic and exocytic traffic.

Similar to mammalian cells, in plant cells, membrane trafficking through the endomembrane system plays essential roles in cell metabolism, growth, and immunity (Kang et al., 2022). The endomembrane system is vital for the transport of membrane-bound cargo both within and outside the cell. Furthermore, plant trafficking pathways enable the building of the majority of the polysaccharide and protein fractions of the CW during various stages of growth. In addition to the plasma membrane (PM), the endomembrane system comprises organelles, such as the endoplasmic reticulum (ER), Golgi apparatus, the trans*-*Golgi network/early endosomes (TGN/EE; herein TGN), and the vacuole (Fig. 1). Plant secretory organelles present unique features that distinguish them from other eukaryotes. For example, unlike mammalian lysosomes, the plant vacuole can occupy a large volume of the cell, pushing the other organelles to the cell periphery. The plant endomembrane system is closely associated with the cytoskeleton, primarily with actin and to a minor but significant extent with microtubules (MT) (Renna et al., 2018; Wang and Hussey, 2015; Hamada, 2014). The cytoskeleton acts as tracks on which organelle and membrane-bound vesicles are transported (Fig. 1). The generation and trafficking of vesicles are tightly regulated by a series of regulators such as small GTPases, tethering factors, and soluble N-ethylmalemide-sensitive factor attachment protein receptors (SNAREs) (Saito and Ueda, 2009; Koike and Jahn, 2022; Kwon et al., 2020).

Both the conventional and unconventional trafficking pathways contribute to membrane trafficking in plant cells (Fig. 1). In the conventional secretory pathway, ER-to-Golgi transport is the first trafficking step. Upon folding in the ER, newly synthesized proteins are assembled into COPII carriers (Barlowe et al., 1994; Brandizzi and Barlowe, 2013; Gomez-Navarro and Miller, 2016). COPII proteins assemble at ER exit sites (ERES) (Sato and Nakano, 2007; Li et al., 2022). From the ER, COPII carriers are transported to the Golgi apparatus for further post-translational modifications (Hadlington and Denecke, 2000). Retrograde transport from the Golgi to ER is facilitated by COPI coat proteins (Zeng et al., 2023). Unlike mammalian systems, a defined ER–Golgi intermediate compartment (ERGIC) is not evident in plant cells, and ERES and Golgi stacks are closely associated, although live-cell imaging analyses have raised the intriguing possibility that the cis-most cisternae of the plant Golgi stacks may share some functions with the ERGIC (Ito et al., 2018). Once secretory cargo has travelled through the Golgi cis-, medial- and trans-cisternae, it is loaded into secretory vesicles at the TGN (Fig. 1). The latter is the major trafficking hub where exo-and endocytic pathways converge (LaMontagne and Heese, 2017). Plant TGNs are highly heterogenous with populations of Golgi-associated TGNs (GA-TGNs) and Golgi-independent TGNs (GI-TGNs) (Rosquete et al., 2018; Uemura et al., 2014, 2019). Plant TGNs can be further compartmentalized with distinct subregions that have been proposed to sort traffic destined either to exocytic secretion or the vacuole (Shimizu et al., 2021) (Fig. 1). From the TGN, cargo is trafficked in secretory vesicles, which can move on the cytoskeleton to eventually fuse with either the PM or to multivesicular bodies (MVB)/late endosomes LE (herein MVB) and then the vacuole (Renna et al., 2018).

Vesicle budding, transport, and fusion are aided by the Ras superfamily of small GTPase-related proteins, which include ADP-ribosylation factors (Arfs), Arf-like, and Ras-associated binding (Rab) proteins (Nielsen, 2020) (Fig. 1). Generally, vesicle budding is determined by the Arf members, while transport and fusion are governed by Rabs and Arf-like proteins. These small GTPases cycle between *ON* (GTP-bound) and *OFF* (GDP-bound) states, acting as molecular switches to regulate vesicle dynamics. The cycling between GTP/GDP-bound states is facilitated by GTPase-activating proteins (GAP) and guanine nucleotide-exchange factors (GEF) (Stenmark, 2009; Borchers et al., 2021). The *Arabidopsis* genome encodes 21 Arf and Arf-like proteins compared with six in mammalian cells and three in yeast (Kahn et al., 2006). The number of *Arabidopsis* Rabs (57) is comparable with mammalian cells (∼60) but much higher than in yeast (11) (Homma et al., 2021; Lachmann et al., 2011), which is indicative of a functional diversification and specialization of Rabs in multicellular eukaryotes. Vesicle fusion to an acceptor membrane is coordinated by the action of SNAREs, Rabs, and tethering factors (Nielsen, 2020; Saito and Ueda, 2009; Kwon et al., 2020). SNAREs are generally transmembrane proteins and classified based on a central amino acid residue of the SNARE motif as either R-SNARE (arginine) or Q-SNARE (glutamine). SNAREs are associated with both the transport vesicles (R-SNARE or v-SNARE) and their target membrane (Q-SNARE or t-SNARE). The interaction between cognate v-SNARE and t-SNARE is required for vesicle fusion (Saito and Ueda, 2009). Upon membrane fusion, SNARE complexes are recycled by N-ethylmaleimide-sensitive factor (NSF) and the soluble NSF attachment protein (SNAP) (Sollner et al., 1993). Aiding the SNARE complex formation and vesicle fusion are multisubunit tethering complexes (MTC) (Chia and Gleeson, 2014). Vesicle fusion to the PM is mainly orchestrated by the MTC, known as the exocyst complex (Žárský, 2022). The exocyst complex was originally identified in yeast and is formed by eight conserved subunits—Sec3, Sec5, Sec6, Sec8, Sec10, Sec15, Exo70, and Exo84 (Mei and Guo, 2018; Saeed et al., 2019). Remarkably, plants have evolved multiple paralogs of exocyst subunits with distinct functions; for example, *Arabidopsis* has 23 paralogs of Exo70 compared with only a single copy of Exo70 in yeast and mammalian cells, while rice has 47 paralogs of Exo70, suggesting an adaptative diversification of the machinery underlying endomembrane functions across kingdoms but also plant species (Cvrčková et al., 2012; Marković et al., 2021). A robust plant immune response relies on rapid internalization by clathrin-mediated endocytosis of surface receptors upon pathogen perception (Ekanayake et al., 2019). To maintain homeostasis, the plant endocytic pathway continuously recycles lipids, membrane proteins, and receptors from the cell surface. Components of this pathway comprise membrane-bound organelles such as EE, LE, and recycling endosomes (REs), which eventually deliver cargo to the vacuole for degradation. Proteins at the PM are endocytosed largely by clathrin-coated vesicles, which fuse to the TGN, an organelle that in plants functions as EE (Tanchak et al., 1988; Dettmer et al., 2006; Viotti et al., 2010). Cargo sorting occurs at TGN, and the proteins are either recycled back to the PM or to the vacuole via MVB (Viotti et al., 2010; Dettmer et al., 2006; LaMontagne and Heese, 2017).

In addition to the conventional membrane trafficking pathway, plants have developed trafficking pathways that bypass the conventional ER–Golgi–TGN route, collectively known as unconventional protein secretion (UPS) (Robinson et al., 2016). Proteins secreted by the UPS lack an N-terminal leader sequence (signal peptide) and their transport is insensitive to brefeldin A (BFA), a drug known to inhibit ER–Golgi traffic (Satiat-Jeunemaitre et al., 1996). As detailed later in this review, the contribution of the conventional trafficking pathway and how it is targeted by pathogens is being characterized, but the role of UPS in the trafficking of defense cargo remains cryptic. Nonetheless, many proteins secreted outside the cell in response to pathogen infection lack a signal peptide (Ding et al., 2012; Krause et al., 2013), suggesting a significant role of UPS in their trafficking that warrants the need for future investigations.

While a mechanistic understanding of the UPS is largely unclear, it is believed that UPS is achieved either by MVB, extracellular vesicles (EV), autophagy, exocyst-positive organelles (EXPO), direct transport at the ER–PM contact sites (EPCS), or a combination of two or more of these routes (Ding et al., 2012; Wang et al., 2010, 2017; Zhou et al., 2022; Robinson et al., 2016). EV are small, membrane-enclosed structures akin to MVB that are released from a cell into the surrounding environment (Zhou et al., 2022). Although the biogenesis and transport mechanisms of EV are not yet known, EV were found to colocalize with MVB at secretion sites. Proteomic analyses of purified EV revealed a cocktail of defense-related compounds, lipids, and RNA (Rutter and Innes, 2017). The EV are secreted to the extracellular space and taken up by fungal pathogens, resulting in a decrease in virulence (Wang et al., 2024). Conversely, pathogenic fungi also release EV, which are internalized via clathrin-mediated endocytosed by plant cells, supporting that EV allow communication between plants and pathogens (He et al., 2023). EXPO is a double-membrane-bound organelle localized at the PM that contains the Exo70E2 subunit but does not overlap with any other known endomembrane markers. EXPO is also not affected by chemical inhibitors of endomembrane traffic, such as wortmannin, BFA, and concanamycin A (Wang et al., 2010). EXPO fuses with the PM and releases single-membrane vesicles into the CW (Wang et al., 2010). EXPO has been implicated in the secretion of arabinogalactan proteins (AGP) (Poulsen et al., 2015), which in turn have been recently found to be important markers for CW defense (Kim et al., 2023), thus linking EXPO with a potential immune-related secretory function. In the packed cytoplasmic environment, organelles interact with each other at membrane contact sites (MCS) (Prinz et al., 2020), and direct secretion from the ER to PM has been proposed to occur via the ER–PM contact sites (EPCS). Interestingly, EPCS rely on the cytoskeleton for the assembly of critical EPCS-resident proteins such as the ER membrane-associated vesicle-associated protein 27 (VAP27) and the actin-associated networked 3C (NET3C) (Hamada et al., 2014). Although the role of plant EPCS in biotic stress has not been addressed yet, the role of VAP27-proteins in virus resistance has been shown (Barajas et al., 2014). In addition, some EPCS proteins localize to the plasmodesmata (PD) (Wang et al., 2016), suggesting a role of EPCS in regulating cell-to-cell traffic, which has a bearing on plant–pathogen responses (Aung et al., 2020). While the role of plant MCS in relation to bacterial immunity has not been defined yet, by being involved in interorganellar communication, MCS may be additional sites for pathogens to disrupt host homeostasis.

### Plant immunity: A sophisticated control of pathogen growth

The CW acts as the first barrier that pathogens encounter and need to overcome to successfully infect their hosts. Most pathogens either reside in the CW-apoplast continuum or actively break the CW for penetration, effectively rendering the CW an active battlefront during infection. Upon overcoming the CW, pathogens encounter the two-tiered plant innate immune system, categorized as pattern-triggered immunity (PTI) and effector-triggered immunity (ETI) (Box 1) (Jones and Dangl, 2006). Plants recognize pathogens by their conserved microbial patterns (microbe-associated microbial patterns [MAMPs]), such as bacterial flagellin, fungal chitin, or peptidoglycans, which are recognized by PM-localized pattern-recognition receptors (PRRs), leading to activation of PTI. PTI activation triggers immune-signaling cascades resulting in defense-gene induction, production and secretion of antimicrobial proteins, generation of reactive oxygen species (ROS), and deposition of callose (β-1,3-glucan) to fortify the CW, aimed at limiting pathogen growth (Ngou et al., 2022; Thomma et al., 2011; Snoeck et al., 2022; DeFalco and Zipfel, 2021). Pathogens overcome PTI by secreting proteinaceous virulence factors (effectors) that target various intracellular components of the host, including transcription factors, cytoskeleton, hormonal pathways, and protein trafficking pathways (Xin et al., 2018; Bhandari and Brandizzi, 2020). Pathogen-elicited effectors dampen immune response using different strategies—slowing down transcriptional reprogramming and the production of antimicrobial proteins, delaying secretion of immune cargo, and affecting interorganelle interactions—while simultaneously promoting nutrient uptake (Tsuda et al., 2009; Mine et al., 2018; Bhandari et al., 2019, 2023; Guo et al., 2016; Kim et al., 2023). Pathogen effectors are in turn recognized by intracellular nucleotide-binding leucine-rich repeat (NLR) proteins, leading to the ETI (Jones et al., 2016). ETI activation leads to the tapping of the same pathways as PTI at a higher amplitude, leading to rapid activation of the immune signaling cascades, hormonal upregulation, and, in some cases, being accompanied by localized cell death, a process known as hypersensitive response (HR) (Cui et al., 2015).

Plants’ readiness to respond promptly to a pathogen attack is significant to determine resistance or susceptible outcomes. However, a hyperactive plant immune system is detrimental to growth as resources are diverted to defense instead of growth. Hence, plants must maintain a balance between growth and defense (van Wersch et al., 2016; Huot et al., 2014). Furthermore, plants have evolved sophisticated signaling systems to minimize ectopic immune activation (Pontiggia et al., 2020; Zhou et al., 2020), which ensured that activation of immunity occurs only upon perception of pathogens and not commensal microbes. An added complexity to plant–microbe interactions is that plants need to maintain a sustainable dose of beneficial microbes to ensure proper growth (Chen et al., 2020; Yuan et al., 2021). Thus, plants need to ensure a measured antimicrobial response that eliminates pathogenic microbes with minimal damage to beneficial microbiota, akin to the human gut (Miyauchi et al., 2023). Plants achieve this by recruiting multiple signaling nodes and trafficking pathways to limit pathogen proliferation (Bhandari et al., 2023; Bhandari and Brandizzi, 2020; Tsuda et al., 2009). The importance of trafficking pathways is highlighted by the dysbiosis of the microbiota in an *Arabidopsis* mutant lacking a key trafficking protein (HopM1-interactor 7, MIN7) and three surface receptors, Flagellin sensing 2 (FLS2), which recognizes bacterial flagellin, elongation factor-tu receptor (EFR), which recognizes bacterial elongation factor, and chitin elicitor receptor kinase 1 (CERK1), which recognizes *Arabidopsis* peptides released upon cellular damage (Chen et al., 2020) and underscores a role of trafficking not only upon immune activation but also in maintaining plant health by assisting in the maintenance of beneficial microbiota.

### The functions of endomembrane organelles in immunity are targeted by pathogens

Coevolution of plant–pathogen interactions has led plants to adopt multiple defense strategies exhibiting functional diversification and partial redundancy (Upson et al., 2018; Derevnina et al., 2021). In turn, pathogens have evolved effectors aimed at avoiding detection, subverting immune pathways, and maintaining a virulent advantage (Yuen et al., 2023; Jeon and Segonzac, 2023). We will take an inside-out view of a cell under pathogen attack, starting from the ER and ending in the extracellular space, and we will discuss how preformed organelles and mechanisms of defense are targeted by pathogens to dampen immunity, and how plants respond to this challenge.

#### Endoplasmic reticulum

**Figure 2.**
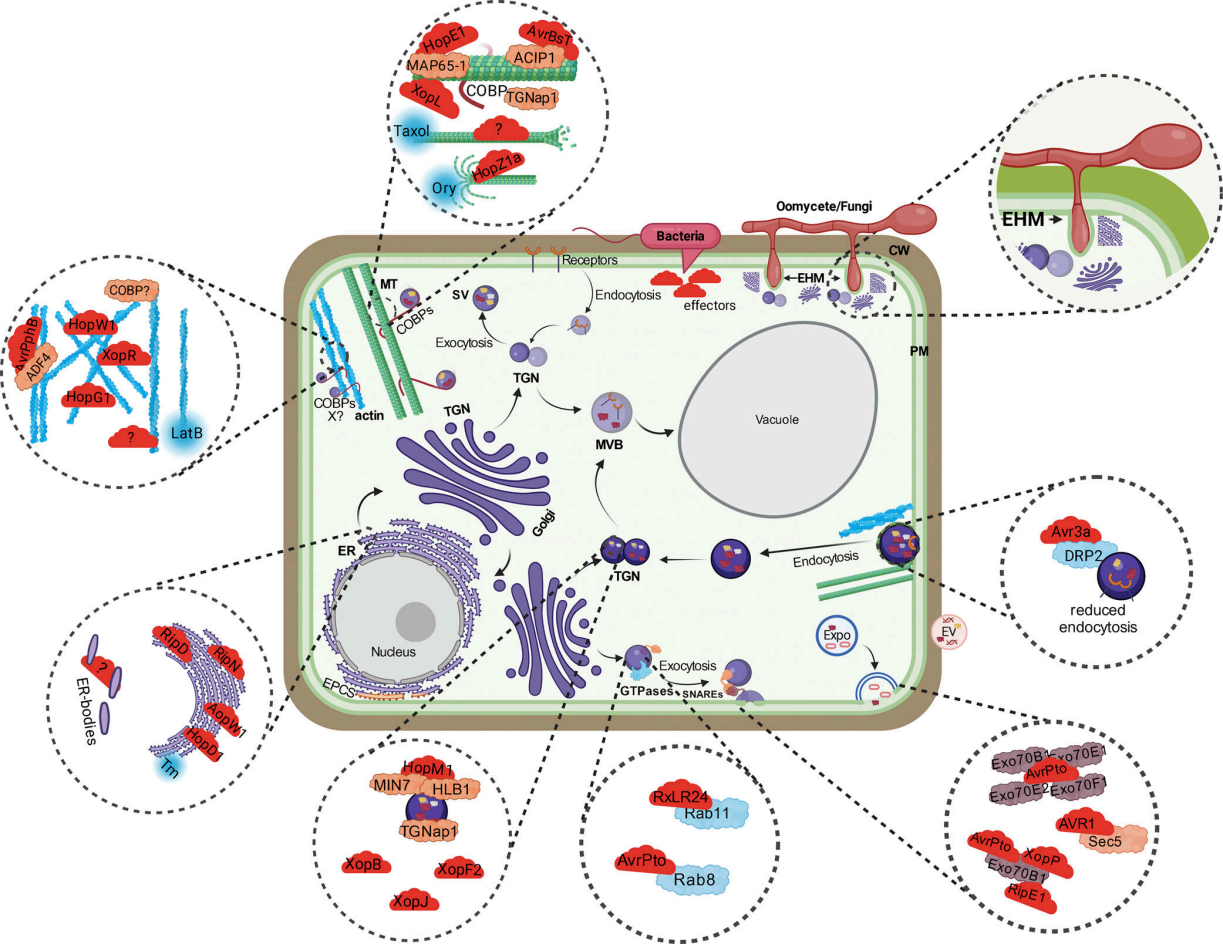
**An overview of pathogen effectors targeting plant trafficking pathways and organelles.** Plant intracellular traffic is coordinated by both the conventional and unconventional trafficking pathways. Pathogen-secreted effectors (red) target multiple host trafficking components including the cytoskeleton, ER, TGN, and small GTPases. The known mechanisms of how these effectors target trafficking-related proteins are illuminated in the main text. In addition to pathogen effectors, various chemicals (blue circles, Ory = oryzalin; LatB = latrunculin B; Tm = tunicamycin) have been instrumental in deciphering the role of trafficking components in immune signaling. Penetration by filamentous pathogens leads to the formation of a plant-derived extrahaustorial membrane (EHM). The EHM surrounds the haustoria acting as an additional barrier. Penetration by pathogens leads to polarized localization of organelles to the site of pathogen penetration, known as focal immunity (Box 2).

Pathogen perception and activation of defense pathways lead to increased synthesis of antimicrobial proteins, which are secreted to the site of pathogen attack (Randow et al., 2013; Brogden, 2005; Lapin et al., 2020). The ER plays a critical role in synthesizing and folding proteins (Strasser, 2018; Phillips et al., 2020). Under stress, the ER increases the expression of proteins, such as ER chaperones and foldases, which can facilitate the folding of secretory proteins (Ko and Brandizzi, 2022; Pastor-Cantizano et al., 2020). Whether under pathogen attack the ER prioritizes the synthesis of defense-related proteins over other secretory cargo is yet unknown. Because the ER is the first step in the synthesis of antimicrobial proteins, it is not surprising that pathogens target the ER to dampen immunity. Upon infection with *Pseudomonas syringae* (*Pst*), a rapid condensation of the ER into “knot-like” structures has been observed (Breeze et al., 2020, *Preprint*). This change in ER morphology was limited to infection with *Pst*, but not with the MAMP flg22, the 22-amino acid peptide of bacterial flagellin, indicating that one or more pathogen-elicited effectors may target the ER. For instance, the bacterial effectors RipD, AopW1, and HopD1 localize to the ER (Jiménez-Guerrero et al., 2023; Block et al., 2014; Jeon et al., 2020) (Fig. 2). HopD1 interacts with the transcription factor NTL9 at the ER, and HopD1 interaction with NTL9 activates NTL9, leading to its nuclear localization (Block et al., 2014). While the mechanisms of how these effectors gain access to the ER remain unknown, a functional Type 3 secreted effector (T3SE) is required for ER remodeling. ER remodeling seems to be a conserved consequence of virulence by both bacterial and fungal pathogens (Micali et al., 2011; Takemoto et al., 2012; Breeze et al., 2020, *Preprint*). A modification of ER organization was observed in the formation of ER bodies upon *Pst* infection (Rufián et al., 2021). ER bodies are large structures formed in the ER lumen characterized by the accumulation of β-glucosidases, and they have been observed in cotyledons and hypocotyls young seedlings in Brassicaceae (Yamada et al., 2008). During *Pst* infection, ER body formation is induced in mature leaves and requires both T3SE and coronatine, but it is inhibited by flg22-induced PTI (Nakazaki et al., 2019; Rufián et al., 2021) (Fig. 2), highlighting the functional interplay between host immunity and pathogen virulence at the ER. RipN, a nudix hydrolase, is a *Ralstonia solanacearum* T3SE, which localizes to the ER and possesses a hydrolase activity (Sun et al., 2019). RipN hydrolase activity reduces the NADH/NAD^+^ ratio without affecting the redox status of the infected tissue. RipN expression leads to suppression of PTI responses such as callose deposition. Therefore, the ER-localized RipN increases pathogen virulence by dampening the PTI response (Sun et al., 2019). The ER monitors the levels of unfolded proteins via the unfolded protein response (UPR) and removes proteins via ER-phagy and the ER-associated protein degradation (ERAD) pathway (Pastor-Cantizano et al., 2020). It has been shown that UPR activation leads to the translocation of the SA-receptor non-expressor of PR1 (NPR1) from the cytoplasm to the nucleus. UPR-driven NPR1 nuclear localization is independent of SA and likely highlights a backup mechanism to ensure the activation of NPR1-dependent immune responses (Lai et al., 2018). While a direct link between UPR and ER-phagy has not been established in plants yet, both pathways contribute to cell death (Yang et al., 2021). Therefore, further studies aimed at elucidating the role of UPR and ER-phagy in immunity will aid our understanding of non-canonical defense pathways recruited by plant immunity.

#### Golgi apparatus and TGN

Differently from the organization of the Golgi stacks in a relatively static ribbon-like structure near the nucleus in vertebrate cells, the plant Golgi stacks are associated with ERES and are highly mobile. Such an organization of the plant Golgi apparatus may allow for bypassing the hindrance of the large central vacuole for cargo delivery and intracellular communication. Similarly, the TGN is highly mobile (Stefano et al., 2015; Rosquete and Drakakaki, 2018). It is therefore not surprising that Golgi and TGN are targeted by multiple pathogen effectors to dampen immune-related trafficking in plant cells (Fig. 2). The most well-known effector targeting a TGN protein is the *Pst* effector HopM1, which binds the TGN-associated protein MIN7 (Nomura et al., 2011). MIN7 is an ARF-GEF necessary for secretion and callose deposition (Nomura et al., 2006). HopM1 binds and degrades MIN7, resulting in increased pathogen virulence (Nomura et al., 2006). The Xanthomonas effectors XopB, XopJ, and XopF2 localize to the Golgi apparatus upon infection (Popov et al., 2016; Priller et al., 2016). While the localization of these effectors and their importance in immunity are documented, their targets are not known yet. It is plausible that effectors targeting Golgi/TGN might adopt one of the following strategies—(i) alter the number and/or the structure of the Golgi/TGN, and/or (ii) slow down anterograde traffic. While a systematic analysis of Golgi/TGN numbers upon infection with pathogens of different lifestyles is missing, the clear increase in secretion of defense-related proteins argues for an increase in the number of Golgi/TGN or at least an increase in anterograde traffic (Uemura et al., 2019). This is also supported by the phenotype of mutants with disrupted Golgi morphology and/or numbers. The barley-conserved oligomeric Golgi (COG) complex, HvCOG3, plays a role in limiting penetration of the powdery mildew causal fungal pathogen *Blumeria graminis (Bgh)* (Ostertag et al., 2013). A loss of COG3 leads to altered Golgi morphology, suggesting a correlation between Golgi structure and secretion aimed at limiting pathogen penetration (Ostertag et al., 2013). The MT-and TGN-associated protein, TGNap1, is involved in the biogenesis of a subset of TGNs (Renna et al., 2018). A TGNap1 loss-of-function mutant, *tgnap1-2*, displays an increase in both the number and size of TGN, indicating a need for TGNap1 in the proper budding of TGN. TGNap1 has been also implicated in both endocytic and exocytic trafficking (Renna et al., 2018). Upon *Pst* DC3000 infection, the *tgnap1-2* mutant is hypersusceptible, and genetic analyses indicated that TGNap1 operates in a pathway distinct from SA to ensure efficient exocytic secretion of antimicrobial proteins (Bhandari et al., 2023) (Fig. 2). Interestingly, TGNap1 is not required for PTI or ETI, but it is specifically required for defense against *Pst* DC3000 but not against the effectorless strain (*Pst* hrcC), suggesting that T3S effectors are necessary to provide a virulence advantage in the absence of TGNap1 (Bhandari et al., 2023). Significantly, TGNap1 and MIN7 were shown to operate additively in resistance to *Pst*, highlighting the functional diversity of immunity-related proteins at the TGN (Bhandari et al., 2023). While effectors targeting MIN7 have been identified (Nomura et al., 2011), it remains unknown if TGNap1 is a direct pathogen target for immune suppression. Further studies would determine if TGNap1 is a direct target and whether pathogen effectors targeting MIN7 also target TGNap1. The heterogeneous nature of the TGN makes it difficult to attribute specific functions or to pinpoint specific effector targets. For example, it is not known if immune activation leads to the recruitment of a specific Golgi/TGN subset for the secretion of antimicrobial cargo or if vesicles are repurposed to fulfill this function. Adding complexity to the role of Golgi/TGN proteins in immunity is the fact that these proteins have both positive and negative effects on immune outcomes. For example, the loss of TGNap1 or MIN7 leads to increased susceptibility, while the loss of Echidna, another TGN-associated protein, leads to autoimmunity (Bhandari et al., 2023; Liu et al., 2022). Nonetheless, the evidence that the Golgi-anchored NLR protein L6 plays a role in flax resistance to fungal rust (Takemoto et al., 2012) but that L6 mislocalization does not affect its effector recognition properties suggests that a Golgi localization of immune targets may not be essential for their function in immunity. These results highlight that effectors may act on targets that are spatially but not functionally associated with endomembranes. In addition, CW-based defenses largely trafficked through the TGN are emerging as essential for limiting pathogen growth (Kim et al., 2023). In the coming years, it will be crucial to identify specific cargo being transported to the apoplast upon infection with pathogens of different lifestyles.

#### Plasma membrane and secretory vesicles

The PM interfaces the inner core of the cell with pathogens and is the site of delivery and fusion of vesicles carrying antimicrobial proteins. The steps of exocytosis and endocytosis must be tightly controlled at the PM, especially during pathogen infection. The integrity of endocytosis involving PM receptors activated by pathogen effectors and their turnover is critical in plant defense (Ekanayake et al., 2023). The TGN-localized clathrin adaptor EPSIN-1 (EPS1) regulates the quantity of FLS2 receptor at the PM, and an *eps1* loss-of-function mutant is impaired in PTI responses (Collins et al., 2020). Similarly, small GTPases also have a critical role in receptor localization at the PM. For example, Rab11 ensures PM localization of the FLS2 receptor (Choi et al., 2013). Furthermore, in yeast 2-hybrid (Y2H) analyses, the *Pst* effector AvrPto interacts with the Rab8, which is required for the secretion of PR1 (Speth et al., 2009), and the *Phytophthora* effector RXLR24 interacts with Rab11 to inhibit PR1 secretion (Tomczynska et al., 2018) (Fig. 2). Similarly, TGNap1, which is required for the trafficking of antimicrobial cargo, including PR1, to the apoplast also interacts with Rab6 (Bhandari et al., 2023; Renna et al., 2018). While a direct role of either Rab6 or Rab8 in immune-related trafficking remains to be tested, these trafficking components might be targeted by pathogens to slow down the traffic of antimicrobial cargo. There are multiple studies attributing also a prominent role of SNAREs in immune processes, including secretion and cementing sites of pathogen penetration and fortification of CW (Baena et al., 2022; Rubiato et al., 2022; Zhang et al., 2007; Kim et al., 2014; Kwon et al., 2008). However, evidence of effector-mediated manipulation of SNAREs is limited. A study based on the screening for interaction between 586 tobacco proteins and 59 RXLR effectors highlighted that the PexRD12/31 family of RXLR effectors could associate with the host’s vesicle trafficking machinery, including SNAREs (Petre et al., 2021), supporting the notion that SNAREs are also targeted by pathogen effectors to subvert immune traffic. Further research is needed to test direct interactions between pathogen effectors and small GTPases and their involvement in immune dampening.

The exocyst complex, which enables the final step of vesicle tethering to the PM, has been involved in PTI, ETI, callose deposition, and exocytosis of defense cargo (Du et al., 2015; Michalopoulou et al., 2022; Wang et al., 2020; Žárský, 2014). In addition to their positive role in plant immunity, exocyst subunits have also been reported to facilitate the growth of the necrotrophic fungus *Botrytis cinerea* (Du et al., 2018). Thus, exocyst mutants can either increase or decrease plant fitness against different pathogens. For instance, plants silenced for the exocyst subunit Sec5 or Sec10 display increased susceptibility to the oomycete *Phytophthora* *infestans* but reduced susceptibility to *B. cinerea* (Du et al., 2018). With its contrasting effects on plant susceptibility, it is not surprising that exocyst subunits are targeted by pathogen effectors. Indeed, the exocyst is targeted by multiple pathogen effectors to hijack one or more of the trafficking roles of this protein complex. For example, the exocyst subunit Sec5 is targeted by the Phytophthora effector AVR1, and this leads to the inhibition of callose deposition and focal secretion of PR1 (Du et al., 2015) (Fig. 2). The AVR1 effector is recognized by the R1 NLR protein, triggering activation of ETI and cell death, suggesting that the exocyst might be guarded by NLR proteins. Furthermore, Exo70, which has the highest number of orthologs amongst the exocyst subunits, seems to be targeted by multiple pathogen effectors. For instance, the Exo70B1 subunit is targeted by the *Pst* effector AvrPtoB, which ubiquitinates Exo70B1 for proteasomal degradation (Wang et al., 2019a) (Fig. 2), and the Ralstonia effector RipE1, a cysteine protease that binds and cleaves Exo70B1 to limit exocytic trafficking (Tsakiri et al., 2023, *Preprint*). In both cases, the Exo70B1 subunit is guarded by an atypical TIR-NBS protein TN2, which recognizes AvrPto and RipE1, triggering HR-like cell death (Wang et al., 2019a). TN2 accomplishes its immune functions by recruiting a class of helper NLRs, known as activated disease resistance 1 (ADR1) (Wang et al., 2021b; Lapin et al., 2020). In addition to AvrPtoB and RipE1, Exo70B1 is also targeted by the Xanthomonas effector XopP. Thus, both Sec5 and Exo70B1 are likely guarded by NLRs. Further studies aimed at exploring the interaction between other exocyst subunits and NLRS would help in identifying if more exocyst subunits are guarded by NLRs and in getting a comprehensive understanding of the immune function of the exocyst. Conversely, a single pathogen effector can target multiple subunits of the exocyst. For example, the *Pst* effector AvrRpm1 indirectly affects Exo70B1, E1, E2, and F1 by interacting with RPM1-interacting protein 4 (RIN4). RIN4, a PM-localized protein, regulates PTI responses in conjunction with the exocyst complex (Redditt et al., 2019). In the absence of a pathogen, RIN4 associates with EXO70 to suppress the secretion of defense compounds. Upon pathogen perception, PTI activation leads to RIN4 phosphorylation, which in turn releases Exo70 from the complex with RIN4 and leads to increased secretion of defense cargo, such as callose (Redditt et al., 2019). PTI activation is suppressed by AvrRpm1, which phosphorylates RIN4 at a site different from the PTI-induced phosphosites, enhancing interaction with Exo70E2 and thereby disrupting exocyst function and secretion (Redditt et al., 2019). Thus, exocyst subunits are involved in a complex interplay at different immune activation layers. *Magnaporthe oryzae*, the pathogen responsible for rice blast, utilizes the effector Avr-Pii, which targets the exocyst subunits Exo70F2 and Exo70F3 (Fujisaki et al., 2015). Thus, multiple effectors of different pathogen origins target the same exocyst subunit. This highlights the convergent evolution of the plant immune system under evolutionary pressure, which likely resulted in plants maintaining multiple orthologs of Exo70. A few pertinent questions remain unanswered; for instance: given that we observe a single effector targeting multiple exocyst subunits and multiple effectors being recognized by a single exocyst subunit, what are the evolutionary advantages and/or recognition trade-offs associated with these interactions? Are pathogens attempting to create multiple points of vulnerability? Would this increase the odds of effector recognition by host proteins? Could it be that different exocyst complexes contain different Exo70 subunits, which are being targeted? And are they functionally diverse? Do the pathogen effectors target the exocyst complex to block either the conventional trafficking pathway or the UPS?

#### Endocytic routes

Similar to exocytic routes, endocytic routes play an important role in ensuring a robust immune response. PM-localized PRRs are constitutively recycled between PM and TGN to ensure their homeostasis (Beck et al., 2012) (Fig. 2). Hence, endocytic pathways are also targeted by pathogens to bypass immune signaling (Ekanayake et al., 2019). The family of dynamin-related proteins (DRP) is involved in endocytosis by facilitating pinching vesicles from the PM. The *P. infestans* RXLR effector (Avr3a) associates with the *Nicotiana benthamiana* protein NbDRP2-1/2, leading to a reduced internalization of the FLS2 receptor (Chaparro-Garcia et al., 2015). AtDRP2A and AtDRP2B are the only known *Arabidopsis* dynamins, with a conserved domain architecture akin to the mammalian dynamins (Ekanayake et al., 2019). Interestingly, both AtDRP2A and AtDRP2B play different roles in PTI. AtDRP2B is required for ligand-induced FLS2 endocytosis (Smith et al., 2014). AtDRP2A is not directly involved in FLS2 internalization but activates selective pathways downstream of FLS2 receptor internalization, such as *PR1* gene induction but not activation of MAPK signaling cascades (Ekanayake et al., 2023). Internalized proteins are sorted at the TGN and MVB. In addition to the TGN-associated MIN7, a recent study implicates another TGN-localized protein, hypersensitive to Latrunculin B 1 (HLB1), in PTI (Nomura et al., 2023, *Preprint*). While our understanding of the mechanisms for PRR activation is extensive, how activated PRRs are internalized is limited. The major roadblocks in our understanding of PRR internalization is likely because of the fast nature of PRR dynamics upon activation, combined with the overlapping nature of PTI and ETI pathways and proteins, and the fact that PTI and ETI pathways are regulated by similar immune signaling nodes.

#### Cytoskeleton

The plant cytoskeleton comprises actin and microtubules (MT) and is essential for transporting antimicrobial proteins and rerouting endomembrane traffic upon immune activation (Li and Day, 2019; Bhandari and Brandizzi, 2020; Li and Staiger, 2018). Thus, it is not surprising that multiple pathogen effectors target the cytoskeleton to slow down traffic and dampen immunity. Pathogen effectors affect the cytoskeleton in two major ways: by directly affecting the homeostasis and architecture of the cytoskeleton, and/or by targeting cytoskeleton binding proteins, thereby indirectly affecting the cytoskeleton organization (Fig. 2). For example, the *Pst* effector HopW1 binds actin directly and disrupts actin leading to reduced endocytosis and increased pathogen growth (Kang et al., 2014). Similarly, another *Pst* effector, HopZ1a, an acetyl transferase, binds and acetylates MT, leading to their depolymerization (Lee et al., 2012) (Fig. 2). While both HopW1 and HopZ1a lead to increased bacterial growth, in contrast to HopW1, which mainly affects endocytic traffic, HopZ1a affects bulk-flow secretion to the apoplast, highlighting the different strategies used by the pathogen to subvert host traffic systems. Direct disruption of the cytoskeleton has a negative impact on plant resistance as supported by chemical treatments to disrupt the cytoskeleton. Oryzalin binds tubulin and inhibits MT polymerization. Plants coinfiltrated with oryzalin and *Pst* DC3000 showed increased bacterial growth, which was dependent on a functional T3SS (Lee et al., 2012; Bhandari et al., 2023). These results underscore the requirement of pathogen effectors to ensure a virulence advantage when the cytoskeleton network is damaged. In contrast, the MT-polymerizing chemical, taxol, had no effect on either host resistance or susceptibility when plants were coinfected with taxol and *Pst* DC3000 (Bhandari et al., 2023), suggesting that, while MT-disruption leads to susceptibility, MT stabilization does not assist plants in resisting pathogens. Latrunculin B (Lat B) is an inhibitor of actin polymerization. The effect of Lat B treatment on plant immunity is not clear with some studies suggesting that it increases pathogen growth (Kang et al., 2014) and others showing that cotreatment of pathogen and Lat B is not significantly different from just pathogen treatment (Leontovyčová et al., 2019) (Fig. 2). Systematic studies are required to establish the role, or lack thereof, of actin-disrupting chemicals with resistance. The cytoskeletal network is maintained by interaction with various accessory proteins, which aid the stabilization of the individual components in dynamic complexes. Actin depolymerizing factor 4 (ADF4) is required for mounting an ETI response to the *Pst* effector AvrPphB (Porter et al., 2012) (Fig. 2). HopG1 interacts with kinesin 7 and induces actin reorganization, which results in leaf chlorosis but not increased pathogen growth (Shimono et al., 2016). Confirming the HopG1-mediated chlorosis, treatment with cytochalasin-D, a chemical that prevents actin filament formation, disrupts the actin network. Coinfiltration of Cyt-D and *Pst* DC3000 leads to increased chlorosis without an increase in bacterial growth. Taken together, these results suggest a connection between actin disruption and symptom formation, although it is not yet known if this actin disruption is beneficial to the plant or the pathogen. Microtubule-associated protein 65-1 (MAP65-1) is an MT-bundling protein targeted by the pathogen effector HopE1 (Guo et al., 2016) (Fig. 2). HopE1 binds MAP65-1 in a calmodulin-dependent manner and dissociates it from the MTs, leading to increased susceptibility (Guo et al., 2016). The Xanthomonas effector AvrBsT, an acetyltransferase of the YopJ effector family, targets MT-localized acetylated interacting protein 1 (ACIP1). During infection, ACIP1 is acetylated by AvrBsT, which modifies its MT binding and causes ACIP1 to aggregate, and this leads to the activation of immunity (Cheong et al., 2014) (Fig. 2). MT-associated proteins are also coopted by fungal pathogens to facilitate penetration of host cells. The fungal *Bgh* protein ROP-interactive protein 1 (ROPIP1) is recruited to the MT by barley MT-associated ROP GTPase activating protein 1 (HvMAGAP1) (McCollum et al., 2020). ROPIP1 recruitment to the MT by HvMAGAP1 leads to MT disruption at the cell cortex and modulates cell polarization in a manner advantageous to the pathogen (McCollum et al., 2020). Though pathogens use multiple strategies to hijack host cytoskeleton-mediated intracellular transport, it is not fully understood if actin/MTs are direct targets of pathogen effectors. The studies discussed above suggest that pathogen effectors target proteins associated with/binding to the cytoskeleton to disrupt trafficking rather than disrupting the cytoskeleton itself. This would be a sound strategy to limit defense response without causing massive cellular damage or cell death. Further studies aimed at understanding the mechanisms and the dynamics of how membrane trafficking is affected during infection may be illuminating. A few pertinent questions may include—whether these effectors attack the cytoskeleton simultaneously or sequentially based on host defense cues; whether pathogen effectors interact with each other to limit damage to the cytoskeleton; and whether pathogen effectors require the host cytoskeleton to translocate within the cell. Additionally, a major gap in the understanding of defense cargo stems from a lack of which proteins are transported in vesicles along the cytoskeleton. As most of the understanding of apoplastic defense is based on the abundance of the PR-family proteins, a more comprehensive understanding of the cytoskeleton-trafficked cargo to the apoplast will provide additional insights into the contribution of actin and MT to immune-related cargo trafficking.

#### Cytoskeleton-organelle bridging proteins (COBPs)

Owing to physical interactions, the spatiotemporal organization of the cytoskeleton largely contributes to organelle and cell morphology. Cytoskeleton–organelle interactions reciprocally determine cytoskeleton organization as well. Thus, the interactions between the cytoskeleton and organelles may dictate various cellular functions and trafficking pathways. Interdisciplinary approaches reveal novel proteins with immune functions. Emergent studies support important roles for proteins that bridge either actin or MT with various endomembrane organelles and play a role in coordinating immune responses. We propose to name these proteins “cytoskeleton-organelle bridging proteins” (COBPs) (Fig. 2). A property of COBPs is that they weakly associate with the cytoskeleton and an organelle but are not integral to the organelle membrane. For example, TGNap1, which lacks a membrane-spanning domain, was identified to have an immune function by coordinating the exocytic trafficking of immune proteins upon infection with *Pst* DC3000 (Bhandari et al., 2023). TGNap1 bridges TGNs and MT and enables the movement of TGN on MT (Renna et al., 2018). Intriguingly, TGNap1 does not have any motor domains, which would be necessary for movement on MT, suggesting that it does not belong to classical motor-domain proteins. However, using a combination of cell biology, proteomics, and chemical approaches, it was shown that the function TGNap1 to traffic antimicrobial cargo is dependent on its ability to interact with MT and TGN (Bhandari et al., 2023). An example of an actin-associated COBP is not yet known; however, HLB1, a protein binding both actin and TGN, has all the hallmarks of a COBP. HLB1 is associated with MIN7-decorated TGN and with actin filaments, and it has been hypothesized to regulate endocytic and exocytic traffic (Sparks et al., 2016). A recent study has implicated HLB1 in immunity (Nomura et al., 2023, *Preprint*). More studies aimed at identifying COBPs involved in immunity will help us expand our knowledge of the plant protein repertoire enabling a robust defense response. This would enable us to identify new pathogen virulence targets that can be engineered for increased resistance and yield.

#### CW fortification

The plant CW surrounds the PM and is made up of a variety of complex polysaccharides chiefly, cellulose, hemicellulose, and pectin. The CW also contains various proteins such as members of the hydroxyproline-rich glycoproteins (HRGPs) superfamily, which provide structural integrity and facilitate cell–cell interactions (Liu et al., 2020). The HRGP superfamily comprises three protein groups: arabinogalactan-proteins (AGPs), extensins (EXTs), and proline-rich proteins (PRPs) (Cannon et al., 2008; Ellis et al., 2010; Albenne et al., 2014; Borassi et al., 2016; Showalter and Basu, 2016). Most CW polysaccharides are either assembled or decorated in the lumen of the Golgi apparatus and transported to the apoplast via conventional traffic. In addition, the plant CW also contains lignin, a highly complex polymer of monolignols containing aromatic compounds connected by a radical reaction. Monolignol is largely synthesized by the phenylalanine pathway which is also important for producing salicylic acid (SA), and the monolignols are transported to apoplast for polymerization most likely via passive membrane transport (Vermaas et al., 2019).

The CW is both the first barrier that pathogens encounter and the last step in defense signaling, which leads to the fortification of the CW (Kim et al., 2023). Most pathogens reside in the CW-apoplast continuum, effectively rendering the CW both an active battlefront during infection and a niche-forming site for pathogens. Moreover, CW breakdown due to pathogen entry or wounding leads to generation of damage-associated molecular patterns (DAMPs), which are recognized by surface receptors leading to activation of PTI-like defense responses, classified as cell wall integrity (CWI) responses (Gigli-Bisceglia et al., 2020). Emerging evidence suggests that CWI maintenance is an integral part of the immune defense (Vaahtera et al., 2019). Moreover, the CW is known to contribute to the functioning of cell surface receptors by restricting their movement or assembly (Martinière et al., 2012; McKenna et al., 2019). Thus, there is feedback between CW arrangement and immune signaling components, which likely modulates immune response. In addition, changes in CW composition, arrangement, or structure adversely affect resistance responses against pathogens (Vogel et al., 2004; Hernández-Blanco et al., 2007; Lionetti et al., 2012; Bethke et al., 2016; Engelsdorf et al., 2017; Molina et al., 2021). Thus, changes in CW, either pre- or post-infection, are consequential for pathogen resistance (Molina et al., 2021; Wan et al., 2021; Dora et al., 2022). Changes in CW composition upon infection most likely serve several purposes, including making space for pathogen infection and growth, providing nutrition to pathogens, and providing signaling cues to modulate pathogen virulence. As a comprehensive review of CW-associated changes upon immune activation is beyond the scope of this review, we highlight here key points of convergence between immunity trafficking and CW.

**Figure 3.**
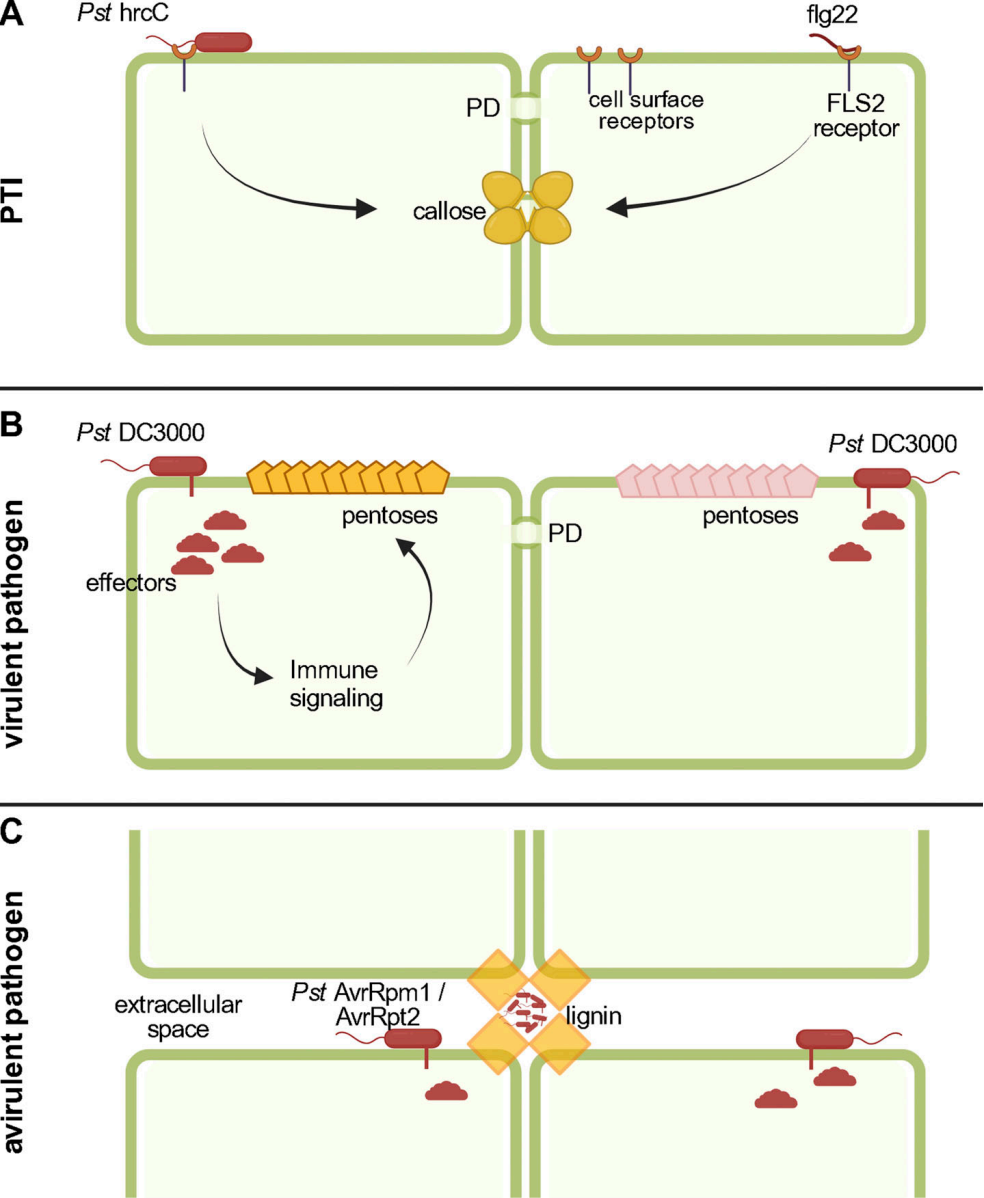
**CW remodeling upon pathogen infection.** The plant CW undergoes extensive remodeling upon pathogen infection and concomitant defense response. The model above highlights the current understanding of CW changes upon infection with different *Pseudomonas syringae* (*Pst*) strains. While many CW changes are likely similar across different *Pst* strains, some CW changes can be used as hallmarks for specific *Pst* strains. **(A)** Callose deposition is a hallmark of pattern-triggered immunity (PTI) elicited by *Pst* hrcC or by other PTI elicitors such as flg 22 or ELF18. Pathogen-elicited effectors target multiple host trafficking processes to dampen callose deposition, enabling the movement of effectors via the plasmodesmata (PD). **(B)** The virulent strain *Pst* DC3000 infection leads to lower callose deposition compared with *Pst* hrcC, as *Pst* DC3000 elicited effectors block callose deposition and in an attempt to open PD. Immune signaling activated as a result of *Pst* DC3000 infection is characterized by the deposition of pentoses (arabinose [orange] and xylose [pink]) and arabinogalactan proteins (AGP). The precise role of these pentoses or AGPs in limiting pathogen growth is yet to be determined. **(C)** Infection with the avirulent strains of *Pst* (*Pst* AvrRpt2/*Pst* AvrRpm1) leads to lignin deposition in extracellular spaces, which encase bacterial cells and limit the spread of infection. Lignin deposition is observed in leaves infected with *Pst* hrcC also, albeit to a lesser degree.

Callose is one of the first macroscopic signs of CW fortification and has been synonymous with CW-related defense (Hauck et al., 2003; Luna et al., 2011) (Fig. 3 A). The timing of callose deposition varies depending on the specific MAMP, indicating that the deposition of callose is a highly regulated and specific process (Luna et al., 2011). Callose deposition also controls PD aperture, which in turn regulates the intercellular movement of signaling molecules and pathogen effectors (Aung et al., 2020). Callose is synthesized by glucan synthase-like (GSL) callose synthases. *Arabidopsis* has 12 callose synthase genes required for various developmental and defense processes. Biotic stress–induced callose deposition is primarily dependent on powdery mildew-resistant 4 (PMR4/GSL5) callose synthase (Jacobs et al., 2003). PMR4 is transported along the conventional trafficking pathway to the PM. Several pathogen effectors target various immune signaling pathways to suppress callose deposition (Iswanto et al., 2022). For example, the bacterial effectors HopM1 (TGN), HopE1 (MT), and XopR (actin) target trafficking components leading to reduced callose deposition (Nomura et al., 2006; Guo et al., 2016). While callose deposition is largely a measure of PTI response, a recent study has identified pentose sugars (xylose and arabinose) and AGPs as markers for CW defense against *Pst* DC3000 (Fig. 3 B). Upon infection with *Pst* DC3000, AGP deposition was focalized to specific CW regions, while in untreated plants AGP deposition was limited to punctate spots distributed in a non-ordered fashion across the CW of mesophyll cells (Kim et al., 2023) (Box 2). Crucially, the level of AGP deposition in the highly crosslinked CW fraction correlates with the duration of infection, reaching a maximum between 48 and 72 h after infection, highlighting the importance of AGPs in CW-based defense (Kim et al., 2023). AGPs constitute a heterogenous family of PM- and CW-localized glycoproteins with redundant functions making them difficult to study (Yates et al., 1996; Tan et al., 2013; Mareri et al., 2019). To date, only the AGP arabinoxylan pectin arabinogalactan protein 1 (APAP1) has been ascribed a role in immunity, as supported by the evidence that an APAP1 knock-out mutant, *apap1-4*, is hypersusceptible to *Pst* DC3000 (Kim et al., 2023). While the mechanisms of AGP deposition in the CW are not yet known, AGPs have been implicated in cellulose synthesis and are likely trafficked through the conventional secretory pathway (Lin et al., 2022). In addition to callose and AGP, lignin is deposited upon immune activation (Lee et al., 2019). In leaves infected with *Pst AvrRpm1* (a *Pst* strain carrying the effector AvrRpm1) lignin deposition is much higher than in leaves infected with *Pst* DC3000 compared with uninfected leaves (Lee et al., 2019) (Fig. 3 C). Confocal microscopy analyses have revealed that bacterial cells of *Pst AvrRpm1* were trapped by lignified cells, indicating that the limited pathogen spread is connected to incompatible interactions by lignin deposition (Lee et al., 2019). Thus, intracellular trafficking pathways play an important role in callose-, AGP-, and lignin-based CW-fortification aimed at limiting pathogen spread in the tissue, although whether these CW modifications are reverted upon elimination of pathogen threat is yet unknown.

### Summary and perspectives

Efficient and timely trafficking of proteins and antimicrobials is essential to fight pathogen infection. The studies discussed here highlight recently identified proteins involved in the trafficking of immune-related cargo and justify renewed interest in better understanding trafficking processes upon activation of immune signaling. The endomembrane organelles and the cytoskeleton enable both activation and execution of immunity. Pathogen recognition leads to global transcriptional reprogramming resulting in increased expression of immune-related genes (Mine et al., 2018; Tsuda et al., 2009). It is likely that, upon pathogen attack, increased transcriptional reprogramming results in immune-related cargo being prioritized for packaging and delivery to limit pathogen growth; however, this is yet to be tested and, if proven true, it would be interesting to establish the underlying mechanisms. Such a goal is complicated by the redundant nature of trafficking proteins and their dual roles in growth and defense, which makes it difficult to attribute specific functions to individual proteins (see Box 3). Several studies have demonstrated that pathogen effectors are useful tools to identify intracellular trafficking candidates. Using the established *Arabidopsis–Pseudomonas* pathosystem, it has been possible to identify some direct host targets of pathogen effectors as evidenced by recent studies revealing immune-related functions of TGNap1 and HLB1 (Bhandari et al., 2023; Nomura et al., 2023, *Preprint*); however, it is likely that other host targets exist. Future studies incorporating a tiered approach of combining chemical perturbation of host processes and pathogen effector mutants are likely to discover new targets and immune-related trafficking components (see Box 3). The field of immunity-driven CW modifications is nascent, but results from multiple labs clearly establish that the CW modifications pre- or post-infection influence plant resistance. While there is clear evidence that different polysaccharides are deposited at the CW depending on the pathogen (Kim et al., 2023; Lee et al., 2019), further studies aimed at cataloging immunity-driven CW modifications and identifying the proteins and trafficking pathways underlying these changes would be illuminating. A spatio-temporal understanding of the CW modifications during immune activation would be also critical to distinguish growth-related CW modifications from the CW modifications tailored for a specific pathogen.

Box 3 Hurdles in identifying and immune-related cargo and potential solutions

Our understanding of plant–pathogen interactions has been enhanced by classical genetic approaches, pathogen effector–host protein interaction studies, and transcriptomic and proteomic studies. We still do not have a comprehensive identity of pathogen-specific immune-related cargo, except for a couple of proteins such as those belonging to the PR family of proteins. A significant hurdle arises from the fact that post-Golgi vesicles have a fast turnover, highly overlapping functions, and do not have distinct markers that can be used to isolate vesicles. This is further complicated by the plant TGN also acting as the early endosome, making it difficult to identify and separate incoming and outgoing vesicles. Nevertheless, a few attempts have been made at identifying post-Golgi vesicle cargo, albeit in non-immune active conditions. For instance, Drakakaki and colleagues have identified the proteome of SYP61-decorated TGN compartment using a combination of vesicle isolation and affinity purification (Drakakaki et al., 2012). The SYP61 proteome contained ECHIDNA, which was eventually characterized to have an immune-related role (Liu et al., 2022), attests to the potential of this approach to identify proteins with novel immune-related roles. Simultaneously, the fact that this approach has not been extensively applied to identify biotic stress-related cargo is not due to a failure of the approach but the technical difficulties in obtaining high-quality vesicle purification from stressed plant tissue. An additional challenge is that immune activation–driven transcriptional reprogramming will lead to overproduction of a few proteins (e.g., PR1, hydrolases), which will lead to a disproportionate representation of these proteins in proteomic studies, making it difficult to identify less abundant but important immune-related cargo. A combinatorial approach combining pathogen challenge (e.g., *Pst* DC3000/*Hpa*) and chemicals that disrupt traffic (oryzalin/endosidin) to identify stalled intracellular immune-related cargo and simultaneously identifying apoplastic proteome would help in deciphering the contribution of a specific traffic route to the trafficking of known immune-related proteins and identification of novel immune proteins. However, this would require a concerted effort across multiple labs with specialization in handling different pathogens.

Another untapped potential avenue of research is in identifying immune-related proteins that are not direct pathogen targets but enable immune-related trafficking functions (for e.g., TGNap1). We propose a forward genetics approach combined with cytoskeletal chemical perturbation to identify proteins that are associated with either actin/MTs and have an immune function. Cotreating mutagenized plant populations with cytoskeleton-disrupting chemicals and pathogens will increase the likelihood of identifying genes involved in the trafficking of immune components is higher. Given the high redundancy in proteins with trafficking functions, narrowing identification to proteins with a role in immunity would be useful.
